# Evaluation of a Novel CLIA Monotest Assay for the Detection of Anti-Hepatitis E Virus-IgG and IgM: A Retrospective Comparison with a Line Blot and an ELISA

**DOI:** 10.3390/pathogens10060689

**Published:** 2021-06-01

**Authors:** Karl Dichtl, Julia Zimmermann, Martin B. Koeppel, Stephan Böhm, Andreas Osterman

**Affiliations:** 1 Chair of Medical Microbiology and Hospital Epidemiology, Faculty of Medicine, Max von Pettenkofer-Institute, LMU Munich, D-80336 München, Germany; dichtl@mvp.lmu.de (K.D.); zimmermann@mvp.lmu.de (J.Z.); 2 Chair of Virology, National Reference Center for Retroviruses, Faculty of Medicine, Max von Pettenkofer Institut, LMU München, D-80336 München, Germany; stephan.boehm@mvp.lmu.de; 3 German Center for Infection Research (DZIF), Partner Site Munich, D-80539 Munich, Germany; 4 Labor Staber & Kollegen, D-81737 München, Germany; m.koeppel@labor-staber.de

**Keywords:** hepatitis E virus, HEV, antibody, monotest, CLIA, LIA, ELISA, serology

## Abstract

Despite the increasing relevance of Hepatitis E, an emerging disease endemic in developing and with increasing numbers of sporadic cases in industrialized countries, commercial tests are mainly based on batch oriented serological assays. In this retrospective study, we compared a line immunoassay (LIA; *recom*Line HEV, Mikrogen) and an ELISA (EIA; Anti-Hepatitis E Virus ELISA, Euroimmun) with a novel chemoluminescence immunoassay in a monotest format (CLIA; Hepatitis E VirClia, Vircell). Twenty sera of PCR proven cases of hepatitis E and 68 blood samples serologically pre-characterized were included. Applying the WHO reference standard, the CLIA demonstrated the highest analytical sensitivity for IgG and IgM. The combinations of CLIA/EIA (IgG and IgM) and CLIA/LIA (IgG) measurements showed substantial correlation. Compared to overall antibody detection (seropositivity in ≥2 assays), CLIA correlation was excellent, outperforming LIA (IgM) and EIA (IgG and IgM). Minor IgM cross reactivity in samples of patients with acute EBV infection was observed in all three assays. The CLIA showed good performance in diagnostic samples compared to established LIA and EIA assays. Due to its ready-to-use monotest format, the CLIA allows simple, time- and cost-effective handling of single samples. These qualities make the assay suitable for diagnostics, especially in the emergency setting and for low-throughput laboratories.

## 1. Introduction

Hepatitis E virus (HEV) is—besides the Hepatitis A virus (HAV)—the most important pathogen of enterically transmitted infectious hepatitis worldwide. From an epidemiologic perspective, hepatitis A seems, at first glance, to outclass hepatitis E with an incidence of more than 100 million cases every year (HEV: 20 million) [1,2]. Nevertheless, hepatitis E is associated with higher morbidity and mortality rates (3 million symptomatic infections and 70,000 fatal courses per year). This demonstrates the significant impact of HEV for health care systems worldwide [1,3].

The four relevant human-transmissible HEV genotypes (GT 1–4) can be divided into two groups with different characteristics, such as their geographical distribution and route of transmission. GT 1 and GT 2 infect several million people each year in Asia and Africa [3]. Both genotypes are transmitted on the fecal-oral route via contaminated drinking water and from person to person under conditions of poor hygiene [4]. HEV seroprevalence rates are particularly high in developing countries ranging from 30 to 80% [5]. The increased perception of sporadic cases of acute hepatitis E in industrialized countries draws attention to GT 3 and GT 4, representing zoonotic transmissions of the virus, i.e., from swine or wild boar: in high-income nations like France, Belgium, the Netherlands or Germany seroprevalence rates up to 30% are described [6,7]. The consumption of insufficiently heated pork meat, e.g., sausages, and contact with infected animals are considered to be the most frequent modes of zoonotic transmissions in high-income countries [8,9]. Human-to-human transmissions play no role for genotype 3 and 4.

After oral ingestion of particles, the virus can cause acute or chronic hepatitis, depending on genotype and pre-existing conditions (i.e., liver cirrhosis or immunosuppression) [10]. Besides the majority of asymptomatic infected, in acutely ill patients there are typical clinical signs of liver inflammation such as fever, nausea, upper abdominal pain and icterus [6]. The course of disease can range from mild infections to fulminant hepatic failure and mortality rates of 0.2–13% [6,11]. Severe disease and fatal outcomes are typically associated with the epidemic genotypes present in developing countries, particularly GT1, with elderly and comorbid patients being at specific risk [6,11]. Symptoms are not different from those caused by other hepatitis viruses or liver damage caused by autoimmune disease [6]. Identifying the causative agent is decisive for therapy, so quick and easy diagnostic tests are required.

Nowadays, a broad range of different diagnostic assays is available that rely either on nucleic acid amplification tests (NAATs) or on immunologic methods. NAAT is a widely used method for diagnosing hepatitis E with the advantage of direct detection of the pathogen. Despite disadvantages like high costs and challenging methodology, the significance of NAATs and particularly PCR continues to grow: for instance, eight European countries have introduced mandatory screening of therapeutic blood products for the presence of HEV RNA by now, as occasional transmission via blood transfusion or organ transplantation has been documented [12,13,14]. Furthermore, in patients under immunosuppression, serologic detection of anti-HEV antibodies may not be as reliable in detecting active infection.

However, the great majority of infections occurs in immune-healthy individuals that mount a strong humoral response. Therefore, HEV infection can be easily diagnosed via antibody detection. With the onset of symptoms, IgM and typically also IgG antibodies against HEV can already be detected in serum. Anti-HEV-IgA assays may be used for further assessment upon suspicion of false-positive IgM results; e.g., due to rheumatoid factor [15,16]. However, interpretation of the results is often still a challenge, as the findings do not appear conclusive and cross reactivities with other viruses may also play a role. Literature mainly identifies polyclonal B-cell stimulation due to acute EBV and CMV infections as cause of cross-reacting IgM antibodies [17,18].

Some epidemiologic studies show a remarkable heterogeneity of IgG seroprevalence depending on the method used, as well as on the population studied [19,20]. A serological reference method is not yet available, but there exists an international anti-HEV antibody standard of the world health organization (WHO).

The knowledge of different virulence of the HEV genotypes is important for epidemiological assessment and prognosis in disease progression, but also for the development and proper selection of diagnostic tools. Several studies indicate that for some commercially available tests, sensitivities for certain genotypes are higher since different antigens of various genotypes are used in the different assays [21,22]. Nevertheless, the existence of only one serotype is still assumed [23]. As many assays have been designed, which rely on GT 1 antigens only, it remains questionable whether these tests can be applied to areas with high GT 3 prevalence.

A broad range of different immunologic assays exists including enzyme linked immunosorbent assays (ELISAs), line immune assays, western blots, and immunofluorescence assays (IFAs), which detect anti-HEV–IgG and -IgM antibodies. Nowadays commercial test formats are typically designed for batch-wise processing, relying on suitable techniques like ELISA. Contrarily, in clinical virology we encounter an increasing relevance of this pathogen in the acute/emergency setting—e.g., diagnostics of acute liver failure. Therefore, assays for easy-to-handle and rapid analysis of single samples are required.

In this study we aimed to evaluate the performance of a novel, CE certified indirect chemiluminescence immunoassay for the detection of IgG and IgM antibodies against HEV, i.e., the Hepatitis E Virclia monotest (VirCell, Granada, Spain; CLIA). The presented results shall help to classify this new assay into the heterogeneity of existing hepatitis E tests. For comparison, we used two tests that have already been characterized in several studies under different conditions [20,24,25]: the *recom*Line HEV IgG/IgM (Mikrogen, Martinsried, Germany; LIA) and the Anti-HEV ELISA (Euroimmun, Lübeck, Germany; EIA).

## 2. Results

**Figure 1:**
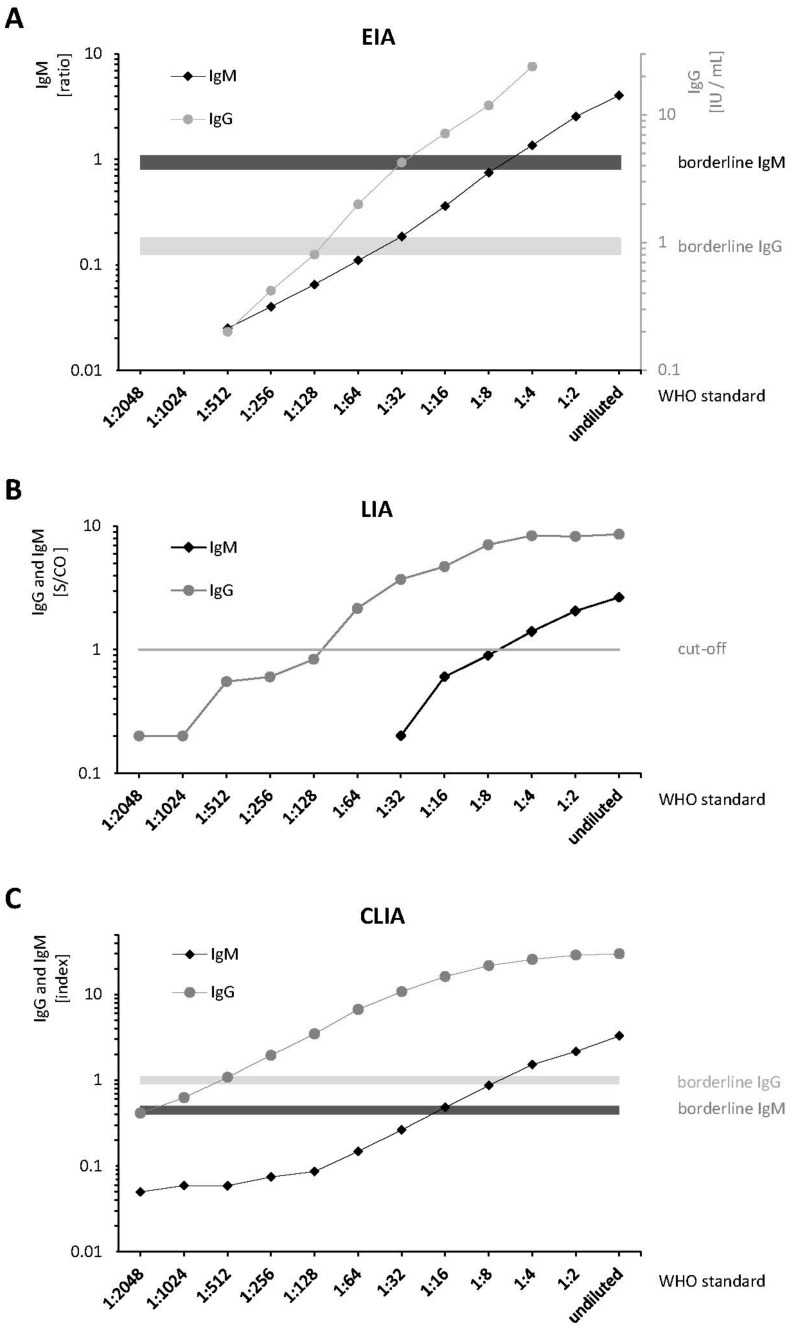
Linearities and detection limits of anti-HEV-antibody assays applied to serial dilutions of the WHO standard. Values indicate the arithmetic mean of two independent runs. The horizontal bars in (**A**,**C**) represent areas with borderline results. (**A**) IU/mL (IgG) and ratio (IgM) cut-off: ≥1.1 = positive; <0.8 = negative (**B**) S/CO band intensity of O2CGt3 cut-off: ≥1.0 = positive; <1.0 = negative (**C**) RLU index IgG cut-off: ≥1.1 = positive; 0.9–1.1 = equivocal; <0.9 = negative; RLU Index IgM cut-off: ≥0.5 positive; 0.4–0.5 = equivocal; <0.4 = negative.

Differences in analytical sensitivities of the three anti-HEV IgG and IgM assays were detected by serial dilutions of the WHO Standard (Figure 1 and supplementary Table S1). Analysis of the serial dilutions showed that the O2CGt3 band of the LIA was always the most intense one. According to the manufacturer, the positivity of this single band is sufficient to obtain a positive test result. Therefore, the O2CGt3 band was used to determine linearity and LoDs. The CLIA demonstrated a higher IgG and IgM sensitivity (≥1 titer level) compared to the EIA and LIA in two independent runs: For IgM, a limit of detection (LoD) of 1:4 was determined using the EIA and the LIA. The CLIA still yielded a positive result for a titer of 1:8. For IgG, the LoD for CLIA, EIA, and LIA was 1:256 (=0.38 IU/mL), 1:64 (=1.56 IU/mL), and 1:128 (=0.78 IU/mL), respectively. Measurement results of the serial dilution demonstrated consistent linearities (Figure 1). Pearson’s correlation coefficients between the provided/calculated and measured concentrations for anti-HEV-IgG and IgM are shown in supplementary Table S1. Notably, the correlation was >0.99 for the anti-HEV-IgG EIA, which is the only assay providing quantitative results based on the WHO reference serum in IU/mL.

In the active hepatitis E subgroup (20 HEV RNA positive cases), all IgM assays demonstrated 100% sensitivity. While the CLIA and the EIA yielded positive IgG results in all samples, the LIA failed to detect IgG antibodies in three cases (Table S2).

**Figure 2:**
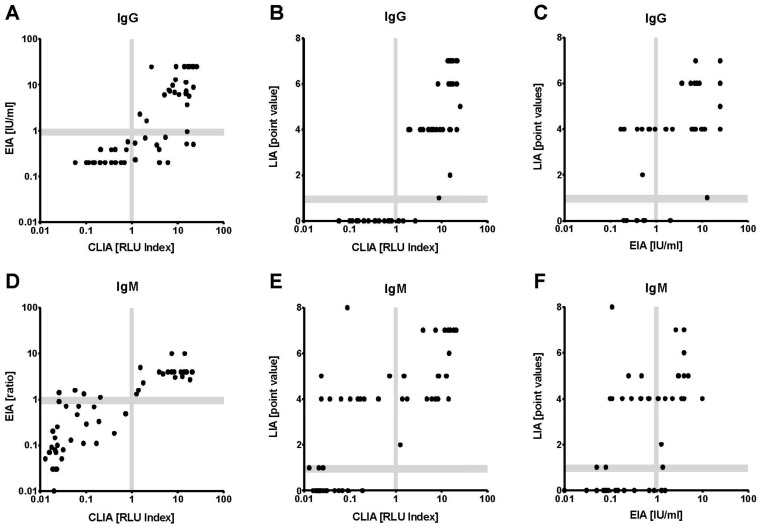
Comparison of measurement results for clinical samples. Fifty-four sera were analyzed applying the three assays. (**A**–**C**) represent IgG measurements, (**D**,**E**) IgM measurements. (**A**,**D**}) compare results from EIA and CLIA, (**B**,**E**) from LIA and CLIA, (**C**,**F**) from LIA and EIA, respectively. Gray bars mark areas with borderline in-terpretation.

**Figure 3:**
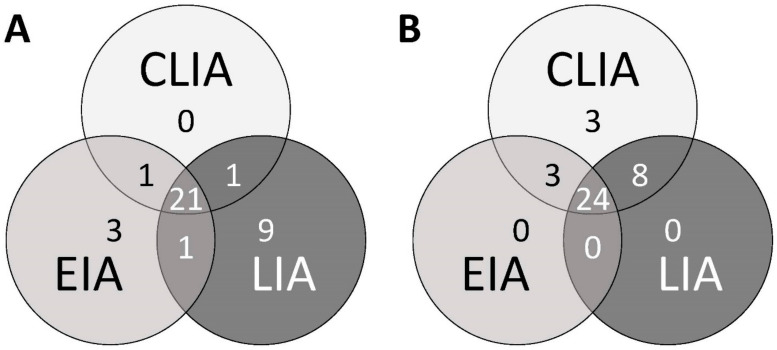
Concordance of positive results. Overlapping areas demonstrate concordance between the different anti-HEV-antibody assays for IgM (**A**) and IgG detection (**B**).

IgG seropositivity rates (all 54 specimens) were 50% for the LIA, 60% for the EIA, and 70% for the CLIA. The correlation of CLIA results and overall IgG (concordance of at least two tests) was excellent (κ = 0.87; Table 1). For the individual assays, CLIA and LIA demonstrated the highest correlation analyzing IgG (κ = 0.76). Not a single CLIA IgG negative serum was tested positive by LIA or EIA (Figure 2A–C and Figure 3B). The three samples tested IgG positive in CLIA but negative in LIA and EIA were characterized by very low CLIA indices close to the cut-off (1.18, 1.20 and 1.50, respectively) (Figure 3B). All respective cases were HEV GT3 infections.

Also for IgM, the results of CLIA and EIA correlated better than in any other test combination (Table 1). There were only four divergent IgM results that measured CLIA negative, but were EIA positive (Figure 2D). These four samples were also measured negative in the LIA (Figure 3A). According to the clinical evaluation reported to the laboratory, the detected reactivities in the IgM EIA in the respective cases was highly likely to be unspecific. Seven of nine CLIA IgM negative and LIA IgM positive sera showed isolated O2CGt3 reactivity in the LIA readout (data not shown). Only one serum was found to be CLIA and EIA positive, but LIA negative (Figure 3A). Regarding the comparison to overall IgM, the CLIA demonstrated the by far highest agreement (excellent correlation with κ = 0.96; Table 1).

Also applying a specificity panel, CLIA and LIA IgG results proved to have good agreement. Ten sera were found to be anti-HEV IgG positive in both tests (Table 2). Only one additional serum with the lowest RLU index in CLIA testing was not detected by the LIA. The IgG EIA yielded positive results in only four sera, all of which were CLIA and LIA positive. This finding might be attributed to the lower sensitivity of the EIA. The positivity rates of 11–32% well reflect the expected seroprevalence in southern Germany [26]. However, the relevance of non-specific IgG reactivity cannot be assessed here. A total of six positive IgM results was generated with the three assays in the EBV subgroup of the specificity panel (Table 2 and Table 3). These were found in four patients. Only one of these patients (patient ID 10) also tested positive for anti-HEV IgG in all three assays. In patients 15 and 18, only the LIA and EIA, respectively, were positive. In patients 10 and 12, always two tests were reactive for anti-HEV-IgM. Overall CLIA, EIA and LIA yielded one, two, and three positive IgM results, respectively.

## 3. Discussion

Current guidelines recommend serology, i.e., analysis of specific IgM and IgG antibodies against HEV, for the diagnosis of HEV infection in immunocompetent individuals [16,27]. For the special emergency setting of acute liver failure, robust, easy-to-use, and fast assays are required. To date, no molecular tests are available that meet all these criteria and a negative PCR does not exclude acute infection [16]. In this study, we analyzed and compared the diagnostic performance of a novel serologic assay, i.e., the HEV VirClia^®^ monotest, with well-established and -characterized tests.

In immunocompetent patients, anti-HEV-IgM is a highly sensitive marker, which is already detectable with the onset of symptoms [22]. Since there is still an unmet need for HEV diagnostics in the acute setting, this study had a particular focus on IgM detection. The highest number of positive IgM results was obtained by the LIA (32 out of 54). These positive results were mainly due to reactivity of the O2CGt3 antigen. However, 8 of these 32 LIA positive results were neither reproduced by the other serologic assays nor by PCR. This raises the need for further investigations focusing on the suitability of this specific antigen [28]. To a lesser extent (n = 3) also the EIA yielded presumably false-positive results. Notably, IgM detection by the CLIA was always supported by at least one additional test (serology or PCR) suggesting the assay did not yield any false-positive results in this study. Furthermore, our results also indicate a high IgM CLIA sensitivity (excellent overall correlation). These findings are in good agreement with the manufacturer’s specifications (sensitivity 95%, specificity 99%). A diagnosis of HEV infection based on a solitary IgM should be verified by either testing of a follow-up sample or NAAT to exclude an unspecific reactivity [16]. Especially in Epstein–Barr virus (EBV) and cytomegalovirus (CMV) infections, false-positive anti-HEV IgM antibodies are frequently detected [17,18]. In our cohort, we also investigated cross-reactivity during acute herpesvirus infections and were able to detect false-positive anti-HEV IgM in all three assays in the subgroup of patients with infectious mononucleosis. Despite the small sample size, it can be estimated that cross-reacting IgM antibodies in the context of polyclonal B-cell stimulation can lead to false positive results in all three assays. Strikingly, three of the four patients had isolated IgM detections without corresponding anti-HEV IgG. This underlines the importance of considering various herpes viral infections as differential diagnosis when selecting serological tests during the work-up of acute hepatitis or elevated liver enzymes. Results should be interpreted with caution and in the context of the IgG and PCR results.

Anti-HEV-IgG is usually detectable with the onset of symptoms as well. In this study, the CLIA was characterized by the highest IgG seropositivity rate, followed by the EIA, and finally the LIA. Generally, differences in test sensitivity depend on a variety of factors including incubation times, secondary antibodies (polyclonal versus monoclonal), and the definition of the cut-off level. However, one major determinant of test performance is the proper selection of antigens to capture the targeted antibodies. HEV serology is typically based on recombinant antigens of ORF2 and ORF3. In particular, the C-termini of the viral proteins encoded in these ORFs have proven to be highly immunogenic [21]. Although only one serotype is postulated for human pathogenic HEV, there is evidence for distinct GT-specific antibody reactivity: various studies from Europe and Asia suggest a significant influence of the antigens applied in the assays on the sensitivity of the respective tests [19,20,29]. Hence, considering the local distribution of HEV genotypes when selecting a test kit is necessary [19,29]. It is very likely that the WHO standard represents the immune response to HEV GT 1, because the hepatitis E of the donor person was acquired in India [30]. In our analysis of the WHO standard, the CLIA, which is based on GT 1 antigen only, demonstrated the highest sensitivity for anti-HEV IgG as well as for IgM compared to the EIA and the LIA, that use both GT 1 and GT 3 antigens.

The WHO standard, actually designed for quantification of anti-HEV IgG, is the serum of a patient with acute hepatitis E [30]. Therefore, it also contains small amounts of anti-HEV IgM. Testing the dilution series of the WHO standard showed good linearities for IgG and IgM in all three assays. The LoDs for anti-HEV IgG and IgM of the EIA were in line with the results of other studies in which assay sensitivity was determined using the WHO standard [24,25,31]. Based on the results of the patient sera analyzed in this study, which also included many GT 3 infections, the CLIA appears to be highly sensitive not only for genotype 1 but also for genotype 3. One could speculate that this high sensitivity results from a decreased LoD compared to EIA and LIA. Thus, the CLIA might detect patients earlier during acute disease and for a longer time period following HEV infection. As a side note, it should be mentioned that the analysis of the c(t) values from the real-time PCR of the twenty RNA positive sera of this study, as well as the comparison with the AST and ALT values measured from these samples, did not allow any conclusions to be drawn about the IgG and IgM positivity rates at different stages of the infection. This might be partly due to the fact that among the patients with low liver values five patients had undergone solid organ or bone marrow transplantation (see Table S2). Therefore, cohorts with longitudinal courses (seroconversion panels) would be desirable in future studies to further investigate the clinical sensitivity of these tests.

Typically, the advantage of higher sensitivity is acquired at the price of lower specificity. However, the extent to which the low CLIA LoD causes false-positive results cannot be precisely determined in our study setting. Large-scale studies are necessary to elucidate the specificity of this novel assay. Nevertheless, in this study no CLIA IgG negative serum was detected positive in LIA or EIA, indicating a high specificity of the CLIA.

The limiting factors in our study are a very small and heterogeneous sample cohort as well as the retrospective study design. This aspect makes a comprehensive, objective comparison of the test systems difficult. The values determined for sensitivity and specificity apply only to the sample cohort of this study and a statement about the true positive and true negative rates remains open. The ready to use monotest strips for processing individual samples have been developed primarily for rare or urgent investigations and serology departments with only few test requests.

## 4. Materials and Methods

### 4.1. Patient Samples

This retrospective study was performed at the Max von Pettenkofer-Institute, which hosts the central virology laboratory for the University Hospital of Ludwig–Maximilians–University (LMU Klinikum) Munich, a 2000 bed university medical center in Munich, Germany. Fifty-four serum samples of individual patients with underlying liver disease, i.e., acute or chronic liver failure, were included in this study. The specimens were sampled in the period of 2005 to 2020 and stored frozen at minimum −20 °C. For the detection of HEV RNA, an in-house PCR was performed, its amplicon sequenced, and genotyped as described previously [21]. Twenty samples were tested positive for HEV RNA (6 cases of GT 1 and 14 cases of GT 3 infection) indicating active hepatitis E. The remaining 34 sera of our in-house HEV assay validation panel had been serologically pre-characterized. However, there is no gold standard for HEV serology. In line with previously published studies, we assumed a positive result (designated “overall HEV IgG/IgM”) by taking into consideration that this is likely when at least two of the three assays yielded a positive test result [25,32]. The in-house specificity panel of 34 sera consists of samples of eleven patients with recent primary CMV infection (defined by positive IgM and low-avidity IgG), twelve patients with typical clinical and laboratory findings (anti-VCA-IgG and -IgM positive, anti-EA-IgG positive and anti-EBNA-IgG negative) of acute EBV infection, and eleven patients with positive detection of anti-HBc IgM in the context of acute or chronic (flare) HBV infection. Analyses for CMV, EBV and HBV were performed using commercial methods according to the manufacturer’s instructions in the accredited routine laboratory of virological diagnostics of the Max von Pettenkofer Institute.

### 4.2. WHO Reference Reagent

The WHO Reference Reagent (WHO standard) for human antibodies to hepatitis E virus (NIBSC Code: 95/584, National Institute for Biological Standards and Control, Hertfordshire, UK) was used to evaluate all three test methods in a standardized manner. The WHO standard was reconstituted according to the manufacturer’s instructions, resulting in an assigned unitage of 100 U/mL. For linearity analysis, the material was serially diluted in anti-HEV antibody negative serum. Test results of the dilution series were determined in two independent runs.

### 4.3. AST and ALT Testing

Measurements of AST and ALT from serum were performed in the routine diagnostic laboratory of the Institute of Laboratory Medicine at the LMU Hospital. Both values were determined using Cobas 8000/c702 (Roche Diagnostics GmbH, Basel, Switzerland) according to IFCC recommendations.

### 4.4. Test Characteristics

Three different CE certified and commercially available serological tests were used for quantitative and semi-quantitative detection of anti-HEV IgG and IgM antibodies. All tests were performed under accredited routine laboratory conditions according to the manufacturers’ instructions.

The Hepatitis E VirClia monotest is a novel chemoluminescence immunoassay test suitable for the VirCLIA monotest system (VirCell) based on the Thunderbolt platform (Gold Standard Diagnostics, Davis, CA, USA). A test strip contains all reaction vessels and reagent containers required for processing; only a wash buffer and aqua dest. must be provided additionally. Like the other two tests, the CLIA works with primary sample tubes, but requires only a minimum of 5 µL serum. The reaction wells of the CLIA are coated with HEV genotype 1 antigen only. Semi-quantitative IgG/IgM results are presented as relative light units (RLU) index.

The Anti-HEV ELISA from Euroimmun is the only commercial anti-HEV ELISA offering a WHO Reference Reagent based quantification of IgG in IU/mL. All test were performed on the EuroAnalyzer II platform (Euroimmun). Semi-quantitative IgM results are presented as ratio. Recombinant HEV proteins of GT 1 and GT 3 are used as antigens to detect antibodies in a minimum of 9 µL serum.

Recombinant HEV antigens (homologues to GT 1 and GT 3 proteins) are applied in the *recom*Line HEV IgG/IgM. They cover different regions of the open reading frame (ORF) 2 (O2N, O2M and O2C) and ORF3 (O3) protein. According to the manufacturer the current evidence is not sufficient to allow “genotyping” with this assay. The interpretation of the results is based on a scoring system (point values) that evaluates the intensities of different bands. An objective evaluation with signal to cut-off measurements (S/CO) of band intensities of the test strips can be performed with the *recom*Scan software. To our knowledge, Mikrogen is currently the only manufacturer to offer a line immunoassay for HEV diagnostics. 20 µL serum per test strip is required.

### 4.5. Statistical Analysis

Statistical analysis was performed using Microsoft Excel 2010 and 2016 (Microsoft Corp., Redmond, USA) and GraphPad Prism 5 (GraphPad Software, La Jolla, CA, USA). Linearity of assays was determined by linear regression analysis using Pearson r correlation function of Microsoft Excel 2016 software. Statistical significance was assumed based on an α-level of 0.05. For comparison of the assays, Cohen‘s kappa coefficient (κ) was calculated and assessed according to Landis and Koch. Values of 0.81–1.00, 0.61–0.80, 0.41–0.60 and ≤0.40 were quoted as excellent, substantial, moderate and poor correlation, respectively [33].

## 5. Conclusions

In this study, the HEV CLIA demonstrated a similar performance in diagnostic samples compared to established serologic assays and excellent IgG and IgM correlation with the WHO standard. Due to the monotest format of the CLIA with ready-to-use reagents including calibrator and negative control, this assay format allows a simple, time- and cost-effective handling of single samples, which is suitable for rapid testing in the emergency setting.

## Figures and Tables

**Table 1:** Correlation of qualitative anti-HEV-antibody results of the three assays. For each comparison, Cohens Kappa coefficient (κ) was calculated.

**IgG**	LIA	EIA	CLIA
EIA	0.60	-	-
CLIA	0.76	0.60	-
overall IgG	0.88	0.71	0.87
**IgM**	LIA	EIA	CLIA
EIA	0.46	-	-
CLIA	0.61	0.75	-
overall IgM	0.64	0.79	0.96

**Table 2:** Positive results in the specificity panel. Sera were obtained from patients with acute infection/flair (CMV, EBV, HBV). Borderline results, which were obtained in one sample by LIA and EIA testing, were not considered for this comparison.

		CMV (n = 11)	EBV (n = 12)	HBV (n = 11)	Total (n = 34)
**IgG**					
	CLIA	3	2	6	11
	EIA	2	1	1	4
	LIA	3	2	5	10
**IgM**					
	CLIA	0	1	0	1
	EIA	0	2	0	2
	LIA	0	3	0	3

**Table 3:** Comparison of anti-HEV IgM positive CLIA, EIA, or LIA results of sera of patients with acute EBV infection. neg. negative, pos. positive.

	IgG			IgM		
Patient ID	CLIA	EIA	LIA	CLIA	EIA	LIA
10	pos.	pos.	pos.	neg.	pos.	pos.
12	neg.	neg.	neg.	pos.	neg.	pos.
15	neg.	neg.	neg.	neg.	neg.	pos.
18	neg.	neg.	neg.	neg.	pos.	neg.
total pos.	1	1	1	1	2	3

## Data Availability

The datasets used and/or analyzed during the current study are available from the corresponding author on reasonable request.

## Supplementary Materials

The following are available online at https://www.mdpi.com/article/10.3390/pathogens10060689/s1, Table S1: Results of anti-HEV-antibody assays applied to serial dilutions of the WHO standard. Table S2: HEV RNA PCR c(t) values, AST, ALT, and LIA IgG results of PCR positive cases.

## Abbreviations

ALT	Alanine Aminotransferase
AST	Aspartate Aminotransferase
CLIA	Chemoluminescence Immunoassay
C(t)	Cycle threshold
EIA	Anti-HEV ELISA (Euroimmun, Lübeck, Germany)
ELISA	Enzyme-linked Immunosorbent Assay
GT 1	Genotype 1
GT 3	Genotype 3
HEV	Hepatitis E Virus
IU/mL	International Units/mL
κ	Cohen’s correlation coefficient
LIA	Line Immunoassay
O2CGt3	C-terminal part of ORF2, Genotype 3
ORF	Open Reading Frame
PCR	Polymerase Chain Reaction
RLU	Relative Light Units
RNA	Ribonucleid acid
S/CO	Signal/Cut-Off
WHO	World Health Organisation
