# Supplementary material for: Evaluation of a Novel CLIA Monotest Assay for the Detection of Anti-Hepatitis E Virus-IgG and IgM: A Retrospective Comparison with a Line Blot and an ELISA
Source: Pathogens. 2021 Jun 1;10(6):689. doi: 10.3390/pathogens10060689 (PMC8228023; doi:10.3390/pathogens10060689)
Supplement: Supplementary file 1 [file pathogens-10-00689-s001.zip › pathogens-1193101-supplementary.pdf]

Table S1. Results of anti-HEV-antibody assays applied to serial dilutions of the WHO standard. Values indicate the arithmetic mean of two independent runs and are given as test specific measurement units. Positive and borderline/equivocal results are depicted in bold and italic, respectively. Considering the sigmoidal saturation curves, correlation was calculated for the linear range, i.e., 1:4–1:512 for IgG and undiluted–1:32 for IgM.

Dilution	EIA IgG [a]	LIA IgG [b]	CLIA IgG [c]	EIA IgM [d]	LIA IgM [b]	CLIA IgM [e]
undiluted	**>25**	**8.60**	**29.82**	**4.05**	**2.65**	**3.25**
1:2	**>25**	**8.25**	**28.82**	**2.55**	**2.05**	**2.15**
1:4	**23.91**	**8.35**	**25.66**	**1.36**	**1.40**	**1.51**
1:8	**11.84**	**7.05**	**21.76**	0.75	0.90	**0.86**
1:16	**7.17**	**4.70**	**16.22**	0.36	0.60	*0.48*
1:32	**4.24**	**3.70**	**10.76**	0.19	0.20	0.26
1:64	**2.00**	**2.15**	**6.66**	0.11	0.00	0.15
1:128	*0.81*	**1.20**	**3.46**	0.07	0.00	0.09
1:265	0.42	0.60	**1.94**	0.04	0.00	0.07
1:512	0.20	0.55	*1.08*	0.03	0.00	0.06
1:1024	n.d.	0.20	0.62	n.d.	0.00	0.06
1:2048	n.d.	0.20	0.41	n.d.	0.00	0.05
**correlation:**						
Pearson (r) *	0.9978	0.9245	0.9131	0.9913	0.9486	0.9781
coefficent of determination (r^2)	0.9956	0.8548	0.8337	0.9826	0.8999	0.9568
rank correlation Spearman (r_{Sp})	1.0000	1.0000	1.0000	1.0000	1.0000	1.0000

[a] IU/mL; cut-off: ≥1.1 = positive (bold); 0.8-1.1 = equivocal (italic); <0.8 = negative; [b] S/CO band intensity of O2CGt3; cut-off: ≥1.0 = positive (bold); <1.0 = negative; [c] RLU index; cut-off: ≥1.1 positive (bold); 0.9-1.1 = equivocal (italic); <0.9 = negative; [d] ratio; cut-off: ≥1.1 = positive (bold); <0.8 = negative; [e] RLU index; cut-off: ≥0.5 positive (bold); 0.4-0.5 = equivocal (italic); <0.4 = negative; n.d. not done.

Table S2. HEV RNA PCR c(t) values, AST, ALT, and LIA IgG results of PCR positive cases. For case B, only a nested PCR but not a real-time PCR result was available. AST and ALT results in U / l. Serology results were only listed for LIA IgG since all other assays yielded positive results in all 20 cases. AST aspartate aminotransferase; ALT alanine aminotransferase.

Case	c(t) Value	AST	ALT	LIA IgG
A	30.0	2116	3392	positive
B	-	489	1137	positive
C	35.0	992	1629	positive
D	29.0	1495	2461	positive
E	26.0	456	641	positive
F	35.0	24	60	positive
G	24.6	79	<5	positive
H	31.4	1547	1930	positive
I	31.0	25	25	positive
J	33.2	153	1004	negative
K	31.8	72	265	positive
L	25.0	4360	4532	positive
M	20.0	1515	3310	positive
N	30.0	2011	1697	positive
O	31.0	103	579	positive
P	31.0	233	823	negative
Q	28.5	1351	2951	positive
R	29.0	127	205	positive
S	36.0	35	45	positive
T	25.8	406	746	negative
